# Designing an effective pulmonary rehabilitation program for severe asthma

**DOI:** 10.3389/fmed.2026.1761011

**Published:** 2026-02-03

**Authors:** Gabriella Guarnieri, Michela Pozza, Andrea Vianello

**Affiliations:** Respiratory Pathophysiology Division, Department of Cardiac-Thoracic-Vascular Sciences and Public Health, University of Padova, Padova, Italy

**Keywords:** aerobic training, asthma exacerbation (AE), maintenance strategies, physical exercise, strength and flexibility training

## Abstract

**Background:**

Despite optimal pharmacological and biologic therapies, many patients with severe asthma (SA) remain symptomatic and functionally impaired, experiencing decreased quality of life and increased healthcare utilization. Whilst physical inactivity can exacerbate symptoms, structured exercise was found to improve functional capacity and immune response in asthmatic patients, highlighting the potential role of pulmonary rehabilitation (PR). The availability of specific, standardized PR protocols for SA patients is nevertheless limited.

**Objective:**

We propose a comprehensive, phenotype-informed PR program tailored for patients with SA. This intervention aims to enhance physical function, optimize symptom control, promote self-management, and foster long-term health behaviors.

**Program overview:**

The A-R-M (assessment-rehabilitate-maintenance) PR model is articulated as follows: multidisciplinary assessment, 8-week rehabilitation phase, end-program evaluation, longitudinal follow-up. Key components include: (1) assessment: baseline evaluations by a multidisciplinary team using validated instruments for quality of life, disease control, exercise capacity, lung function, mood, and educational needs; (2) rehabilitation (education + exercise): education: eight sessions covering topics such as asthma pathophysiology, pharmacological treatment, self-management strategies, nutrition and satisfaction assessment. Exercise prescription: an individualized, physiotherapist-supervised regimen incorporating aerobic exercise, strength training, and inspiratory muscle training; and (3) maintenance and monitoring: personalized plans to support sustained physical activity and behavior change. Expected outcomes include significant improvement in exercise capacity, symptom control and quality of life, along with reduced use of oral corticosteroid, exacerbation's numbers and unplanned specialist visits.

**Conclusion:**

The A-R-M program offers a practical, evidence-based approach to improve outcomes of SA patients by addressing symptoms, function, and self-management, ultimately reducing healthcare costs and enhancing patients' wellbeing.

## 1 Background

Asthma is a chronic inflammatory respiratory disease with a steadily increasing global prevalence. Symptoms and airflow limitation fluctuate in intensity and frequency over time and can be triggered by irritants, physical exercise, or viral infections (1). In patients with severe asthma, disease control often remains suboptimal despite maximal inhaled therapy and additional medications, leading to higher morbidity, mortality, and a substantial impact on quality of life (QoL) (2). Although advances in pharmacological and biologic therapies have improved outcomes, a considerable proportion of patients remain symptomatic, underscoring the need for complementary non-pharmacological interventions (1).

Patients with severe asthma tend to be less physically active than healthy individuals or those with mild to moderate disease, as symptom exacerbation during exercise often leads them to avoid physical effort (3). Conversely, in severe asthmatic patients higher levels of physical activity are associated with a lower risk of exacerbations, fewer symptoms, a slower decline in lung function, and reduced bronchial hyperresponsiveness, suggesting a potential anti-inflammatory effect (4).

Pulmonary rehabilitation (PR) is an adjunct to pharmacologic treatment aimed at improving the physical and psychological condition of patients and promoting the long-term maintenance of healthy behaviors, including a more active lifestyle (5). To date, the evidence of PR efficacy is heterogeneous and limited in severe asthma (5, 6). A structured program that combines exercise training, education, and maintenance is expected to yield clinically and functionally meaningful benefits while also representing a cost-effective intervention (7).

There is a need for guidance that adopts a personalized, phenotype-informed approach to rehabilitation referral and programs, with early identification of severe asthma patients who are deconditioned, physically inactive, or experiencing frequent exacerbations despite optimal pharmacotherapy. Multidisciplinary teams should tailor interventions to address comorbid contributors such as obesity, psychological distress, and sinonasal disease, and PR programs should incorporate objective activity monitoring, stratified goal setting, and family or community support to promote long-term behavior change. Programs should also consider occupational and social determinants of health and offer flexible scheduling and hybrid delivery models. Safety screening, optimization of inhaler technique, individualized action plans, and integration with severe asthma clinics are also essential components.

We propose an integrated gradual rehabilitation program based on current evidence, including moderate-intensity exercise tailored to the individual according to the FITT-VP principle (frequency, intensity, time, type, volume, progression), a multidisciplinary eight-session educational course on distinct topics, and a maintenance program.

## 2 PR program

Eligible patients for PR program are those with severe uncontrolled asthma, treated in GINA 4/5, with priority given to those who are deconditioned and physically inactive. According to ATS/ERS definitions (2), severe asthmatics are patients in which the disease remains uncontrolled despite adherence to maximum-dose ICS and long-acting beta2-agonists (LABA) or worsens when the treatment dose is reduced. The assessment of asthma control is based on: asthma control questionnaire (ACQ) score consistently ≥1.5, or an asthma control test (ACT) score <20; frequent severe exacerbations, defined as ≥2 courses of systemic corticosteroids in the previous year; severe exacerbations, defined as ≥1 hospitalization in the last year; a predicted post-bronchodilator FEV_1_ < 80%, in the presence of a reduced FEV_1_/FVC ratio, defined as below the lower limit of normal (2). Patients are defined deconditioned and physically inactive according to a level of exercise tolerance below the lower limits of normality (LLN), expressed in meters walked and based on the reference equations of Enright and Sherrill (8).

**Figure 1:**
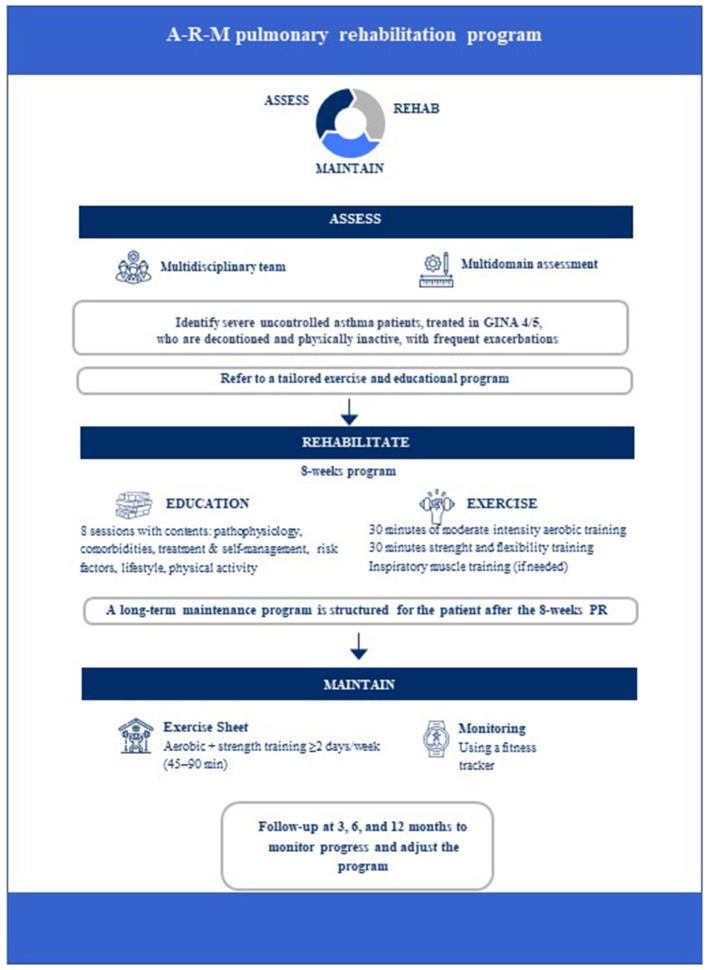
A-R-M pulmonary rehabilitation Diagram with summary of phases of program.

In accordance with the 2015 ATS/ERS Statement, the proposed PR program is structured into four phases: an initial multidisciplinary assessment, an 8-week rehabilitation phase, a final evaluation, and follow-up and it is managed by a multidisciplinary team (7). The team was selected based on the characteristics of patients with severe asthma and their comorbidities and may vary according to specific cases and availability. Basically, the group of specialists is made up of the pulmonologist, who knows and treats the patient, the cardiologist who may support exercise testing and the management of cardiovascular comorbidities, while a physiatrist may be involved in prescribing assistive devices and supporting the physiotherapist in planning individualized rehabilitation interventions (6). In Figure 1 we summarized the phases of the program that we have defined “A-R-M” (Assessment, rehabilitate, maintain) pulmonary rehabilitation program.” The program is organized into four phases: assessment, educational and exercise program as part of rehabilitation, and mainteinance. The phases are detailed below.

### 2.1 Assessment

The initial assessment is conducted as part of a first visit at a multidisciplinary outpatient clinic, whose staff includes one pulmonologist, one cardiologist, one physiatrist, and one physiotherapist. A comprehensive rehabilitation record is developed, incorporating all necessary indicators for the formulation of a personalized rehabilitation program, specific measures to be collected at the end of the program, as well as during follow-ups at 3, 6, and 12 months. The selected measures cover various domains: quality of life (Mini-AQLQ, SF-36); symptoms (ACT, ACQ); exercise performance (6MWT), handgrip and quadriceps strength assessed via dynamometers, and CPET; physical activity (IPAQ-SF); respiratory function (spirometry values, FeNO, MIP, and MEP); body composition (BMI); anxiety and depression (HADS); and educational needs (LINQ). Additional metrics include the frequency of exacerbations, hospitalizations or unscheduled visits, and other functional limitations pertaining to activities of daily living (7, 9).

### 2.2 Education

The educational program is defined according to existing literature (1, 7, 10, 11): (a) number of sessions: 8; (b) duration: 45 min per session (webinar) or 1 h (in-person); (c) delivery format: 6 sessions provided as recorded webinars, including presentation slides utilized by the speaker, supplemented by 2 in-person meetings—the first and last sessions—with opportunity of remotely participation; (d) multidisciplinary team, consisting of the following healthcare professionals: 1 pulmonologist, 1 cardiologist, 1 physiotherapist, 1 dietician, and 1 psychologist; and (e) content areas: asthma pathophysiology and comorbidities; asthma treatments and self-management strategies; management of asthma risk factors; dietary considerations; physical and mental wellbeing; and the role of physical activity and exercise. A multimodal and multisensory instructional approach is adopted, ensuring a combination of audio, visual, and experiential learning components. Participants are empowered to select content independently. Each educational session is accompanied by a brief satisfaction questionnaire to evaluate the usefulness, clarity, ad effectiveness of the material presented. The questionnaire can be completed online through Google Forms. The Educational Program is detailed in Supplementary Table A.

### 2.3 Exercise

The exercise program is tailored to the patient according to initial assessment and is supervised by a physiotherapist. Setting: outpatient + home-based; duration: 8 weeks; weekly frequency: three sessions (two supervised and one unsupervised home-based). Delivery mode: consists of individual supervised or group sessions, with suggestion for at least one additional unsupervised home-based session weekly. Session duration: each session lasts 1 h.

#### 2.3.1 Supervised exercise program

a) Aerobic training: involves 30 min of moderate-intensity aerobic exercise performed on a cycle ergometer at 40%−69% of VO_2_ max or 55%−74% of HR max. Each session will comprise a 5-min warm-up, followed by 20 min of exercise at a specified heart rate, and concluded with a 5-min cool-down. Aerobic training can be modified from continuous to interval formats, according to the patient's clinical characteristics. Reference parameters will be established based on the CPET performed during the assessment phase (12–14).
b) Strength and flexibility training: the exercise regimen is customized according to individual characteristics identified during the initial assessment and is adjusted for functional limitations and comorbidities. Exercises may involve both single-joint and multi-joint movements, executed unilaterally or bilaterally, targeting one or more muscle groups, with or without external resistance. Resistance will be maintained at 60%−70% of the individual 1-RM, calculated using the Epley formula (15). Exercises will comprise 8–12 repetitions over 1–3 sets, with rest intervals between 1 and 3 min. The overall duration of this segment is 30 min (12–14). Progression will be determined based on 1-RM assessments and the Borg scale ratings during exercise execution. If scores fall between 12 and 14, they will be maintained; if lower, the intensity can be increased; if greater than 14, one of the parameters (repetitions, intensity, or load) will be decreased (16).
An appropriate breathing technique should be maintained throughout all exercises. Example of a possible exercise program is reported in Supplementary Table B.
c) Inspiratory muscle training (IMT): this program is designed for patients who exhibit significantly reduced inspiratory and expiratory muscle strength, characterized by PiMax and PeMax values below 50% of the predicted values during the initial evaluation (17). The IMT regimen consists of 30 min of training per day, 6 days a week, over a duration of 6 weeks. Patients receive instruction for the use of the training device during individual sessions and will be provided with a home-based training program. At each follow-up session, the correct utilization of the device will be assessed, and the resistance load will be adjusted as necessary. Respiratory muscle training will be conducted at ≥30% of the baseline measured PiMax, employing the Threshold IMT device.

#### 2.3.2 Unsupervised home-based exercise program

Exercises performed at home are unsupervised. Patients are encouraged to complete at least one session per week independently; however, there is no contraindication to performing additional sessions. The home-based training adheres to the same principles as the supervised sessions but is tailored to the individual's social and environmental context, as well as the availability of equipment. Safety during exercise execution must always be ensured. Throughout the program, changes to the exercise regimen will be made based on the patient's progress. Each patient receives a personalized exercise handout that includes specific instructions for execution, prescribed repetitions, and illustrative images to facilitate correct performance.

a) Aerobic activity: patients are instructed to perform 30 min of stationary cycling (if available) or treadmill exercise. Alternatively, brisk walking is encouraged, guided by the Borg Rating of Perceived Exertion (RPE) scale; whenever possible, heart rate monitoring should be utilized. Walking circuits that can be completed indoors may also be recommended.
b) Strength and flexibility training: a personalized home-based routine will be provided, based on exercises performed during supervised gym sessions. Small equipment used in the gym may be replaced with household items (e.g., plastic bottles can serve as substitutes for dumbbells).

Treatment adherence will be monitored through a diary or log sheet maintained by the patient, recording home-based sessions and any symptoms that may arise during exercise performance. Where feasible, the use of a fitness tracker is advised to enable more precise monitoring of exercise activity.

### 2.4 Maintenance

Maintenance program is structured to sustain the exercise regimen prescribed during the rehabilitation phase, adapting it to the socio-environmental resources available to the patient. Each participant will receive an exercise sheet that reports the skills learned during the rehabilitation process and the progress achieved. To monitor physical activity levels—including sedentary time and active time—the use of a fitness tracker is recommended, particularly during the follow-up period. Adjustments to the maintenance plan will be proposed at each scheduled follow-up visit, and during any unscheduled consultations the patient may request. Engagement in aerobic activities and strength training exercises at least 2 days per week is encouraged, with session durations ranging from 45 to 90 min. Additional moderate-to-vigorous physical activities may be undertaken, consistent with the presence of respiratory symptoms.

## 3 Expected results

The exercise component is expected to improve muscle strength and endurance (18–21), alleviate asthma symptoms (18, 19, 21–23), improve quality of life (18, 21), and reduce the frequency of exacerbations (21, 24) (see Table 1). While few studies have investigated its effects on immunomodulation and inflammation in patients with severe asthma, findings from research focusing on mild to moderate asthma are promising (25). Indeed, exercise and comprehensive PR programs may exert favorable immunomodulatory effects, decreasing airway and systemic inflammatory markers (including FeNO, sputum eosinophils, and selected pro-inflammatory cytokines), reducing airway hyperresponsiveness and attenuating the frequency and severity of exacerbations through improved airway clearance, autonomic balance and systemic metabolic health (26, 27).

**Table 1:** Expected clinical and functional improvements following the application of A-R-M pulmonary rehabilitation program.

**Outcome (measure)**	**Expected enhancements**	**Evidence based justification**
Asthma control (ACQ)	Clinically significant improvement (MCID = 0.5)	−8 weeks program (19) - Frequency: 3/weeks (19) - Difficult-to-treat asthma (19) and severe asthma (18, 22) - Physical activity and aerobic exercise (21, 23), moderate intensity exercise (18), exercise training combined with educational program (19) - IMT could improve dyspnea and other symptoms (17)
Quality of life (AQLQ)	Clinically significant improvement (MCID = 0.5) in at least one of these domains: symptoms, activity limitations, emotional function.	- Physical activity combined with other interventions (21), moderate intensity exercise (18) - IMT could overall improve quality of life (17, 32)
Lung function (Spirometry)	Improvement in pre-post intervention in at least one of these parameters: FVC, FEV1, PEF, FEV1/FVC	- Aerobic exercise (21) and educational programs (24) (FEV1) - IMT could improve dynamic lung volumes (17, 32) - Physical activity (21)
Exercise capacity (6MWT)	Significant improvement in walking distance, not clinically significant (MCID = 30 m) Improvement in dyspnea and symptoms observed during the test.	−8 weeks program, frequency: 3/weeks, difficult-to-treat asthma (19) - Physical activity (20), Physical activity combined with other interventions (21), moderate intensity exercise (18) - IMT could improve distance and symptoms during the test (17, 32)
Anxiety and depression (HADS)	Improvement in pre-post interventions, but not clinically significant difference (MCID = −1.7/−1.5)	- Educational programs (41)
Exacerbations (number)	Percentage reduction in the number of asthma exacerbations compared to the year before the PR program	- Educational programs (21, 24) - 8 weeks program, frequency 1/week (1 h exercise, 1 h education) + 2 home based sessions; contents: Muscle resistance, aerobic training, educational program (10)

ACQ, asthma control questionnaire; AQLQ, asthma quality of life questionnaire; MCID, minimal clinical important difference; 6MWT, 6 min walking test; HADS, Hospital Anxiety and Depression Scale; IMT, inspiratory muscle training.

Additional improvement can be expected in clinical parameters, treatment adherence, and participation in activities of daily living (ADLs) (22), alongside a reduction in the incidence of exacerbations (21, 24). Modifications in body composition as a result of the intervention are also awaited (18, 19). It is important to underline that benefits achieved can be sustained over the long term, although a potential decline may occur between the immediate post-intervention assessment and the 12-month follow-up. Nevertheless, improvements from baseline are expected to persist, supported by structured maintenance program and supervised follow-up evaluations (7, 19).

## 4 Discussion

Severe asthma treatment still represents a major challenge, despite advancements in pharmacological therapies. The evidence regarding PR in asthma is heterogeneous, and the study populations are small, weakening the strength of recommendations and limiting their generalizability (5). Originally implemented in chronic obstructive pulmonary disease (COPD), PR has been progressively extended to other respiratory conditions; however, switching COPD methodologies to asthma may yield misleading results (28, 29).

Current evidence suggests that structured exercise, particularly when supplemented with education and behavioral support, can improve exercise capacity, reduces dyspnea, enhances health-related quality of life (HRQoL), and mitigate exacerbation burden in selected cohorts (19, 21, 23, 30, 31). Inspiratory muscle training (IMT) offers significant improvements in inspiratory strength and symptom relief, especially when respiratory muscle weakness is present (17, 32). In patients with comorbidities, PR may provide additional indirect benefits, such as weight reduction (10, 18, 19), fall prevention (33), and support for smoking cessation. Education is known to influence knowledge, perceived benefits, health beliefs, behaviors, and clinical outcomes (34). Moreover, educational programs can reduce exacerbation rates and enhance treatment adherence, as well as contribute to improvements in lung function and exercise capacity (24). For long-term maintenance, adherence and motivation are critical (6). Factors such as self-efficacy, awareness of benefits, and variety in exercise regimens promote adherence, while time constraints and fatigue pose significant barriers (35). Family involvement and broader patient awareness can further reinforce adherence (36, 37), whereas group activities foster peer support and a sense of community (38).

To our knowledge, no prior studies have provided a detailed description of the components of an integrated outpatient pulmonary rehabilitation program specifically designed for severe asthma. The study of McDonald et al. (39) represents an excellent reference for patient assessment and multidisciplinary management; nevertheless, it did not provide a detailed exploration of the specific exercise modalities, educational content, or maintenance strategies included in the intervention. In contrast, Schultz et al. (40) provided a detailed description of the program, but it was delivered in an inpatient setting and therefore differs in terms of setting, costs, and modes of delivery. Other studies have addressed pulmonary rehabilitation in asthma, although they were not specifically tailored to individuals with severe asthma (26). Our A-R-M PR program does not propose a novel intervention; rather, it synthesizes the best available evidence and adapts it for practical application. The integrated and multidisciplinary approach provided by our program may constitute an effective strategy for addressing the underlying mechanisms of exacerbations by targeting multiple dimensions—inflammatory, functional, and self-management—while simultaneously alleviating the burden of comorbidities and systemic consequences associated with asthma. As a result, the need for rescue medications, additional corticosteroid treatments, urgent specialist consultations, and hospital admissions can be decreased.

PR stands out as one of the most cost-effective interventions for chronic respiratory diseases, with a cost-benefit profile more favorable than pharmacological treatments such as tiotropium, long-acting beta-agonists (LABAs), triple inhaled therapy, or telehealth services; indeed, it ranks just below influenza vaccination and smoking cessation. The estimated cost for PR ranges from £2,000 to £8,000 per Quality-Adjusted Life Year (QALY), that is significantly lower than £7,000 to £187,000 per QALY associated with triple inhaled therapy (7). Improvement in comorbid conditions, such as a reduction in body mass index (BMI), can lead to further reduction in direct and indirect healthcare costs (22, 23). The effectiveness of non-pharmacological interventions in the contest of a treatment decision pathway may prevent escalation to higher treatment tiers, including the initiation of biologic therapies, in patients who are not yet receiving such treatments (1, 7). Long-term retention of achieved improvements is expected to result in decreased economic expenditure (7).

## 5 Conclusion

This study examined the planning of a PR program for patients with severe asthma. We designed its structure, components, and supporting materials, achieving our main goal of providing a theoretical model ready for pilot testing. Several limitations should be acknowledged. As a pre-implementation study, no clinical data concerning its application are yet available. This theoretical phase was nonetheless necessary, given the program's complexity. In addition, the content of the program was affected by the variable quality of evidence and the absence of standardized referral criteria.

Of importance our A-R-M PR program not only proposes a theoretical model but also presents a practical framework to address an unmet care gap. Through sustained and collaborative efforts, this model can be translated into clinical practice and facilitate meaningful improvements in care, empowering individuals with severe asthma to achieve better outcomes and enhanced quality of life. Funding should prioritize pragmatic trials and implementation of research in the area of PR, and healthcare systems should adopt reimbursement mechanisms to support multidisciplinary rehabilitation teams. Ultimately, incorporating PR into the strategic framework of Severe Asthma Centers may optimize patient-centered outcomes and reduce healthcare utilization.

## Data Availability

The raw data supporting the conclusions of this article will be made available by the authors, without undue reservation.
