# Supplementary material for: Designing an effective pulmonary rehabilitation program for severe asthma
Source: Front Med (Lausanne). 2026 Feb 3;13:1761011. doi: 10.3389/fmed.2026.1761011 (PMC12909588; doi:10.3389/fmed.2026.1761011)
Supplement: Supplementary file 1 [file Table_1.docx]

**Supplemtary table A**: **Educational Program illustrating the main fields of interest, contents and materials, application methods and the professional figures involved**

| **TITLE** | **CONTENTS** | **TARGET AUDIENCE** | **MATERIALS** | **TEAM** |
| --- | --- | --- | --- | --- |
| **1- Welcome to the pulmonary rehabilitation program** | The session provides an overview of:   - Delivery methods - Duration and frequency - The multidisciplinary team - Goals and benefits of the program   It also offers guidance on:   - How to manage emerging symptoms - How to contact and interact with the care team during the program   Ample time is dedicated to addressing questions and concerns from patients and their family members. | Patient,  Caregivers,  General Practitioner (GP) | - Presentation slides - Recording of the session - Short informational brochure (both printed and available online) | Pulmonologist,  Cardiologist  Physiotherapist, Dietician,  Psychologist |
| **2- Understanding Asthma and Its Comorbidities** | Definition of Asthma and Its Clinical Manifestations  The session provides a definition of asthma and its key clinical features. It offers an in-depth look at symptoms that are particularly relevant to rehabilitation, such as dyspnea and bronchoconstriction, and focuses on the concept of asthma control.  It also highlights complications and comorbidities that may negatively affect asthma and its management. | Patient,  Caregivers | - Presentation slides - Recording of the session | Pulmonologist  Cardiologist |
| **3- Asthma treatment and Self-management** | Current Therapeutic Pathways for the Treatment of Asthma and Its Symptoms.  The session explores both pharmacological and non-pharmacological treatments for asthma.  Particular attention is given to the individualized asthma action plan, along with the description and promotion of self-management strategies. | Patient,  Caregivers | - Presentation slides - Recording of the session | Pulmonologist |
| **4- Risk factors** | Asthma Risk Factors and Management Strategies  The session describes the main risk factors for asthma and its exacerbations. Practical guidance is provided on how to manage these risk factors, such as exposure to allergens or cigarette smoke.  Direct contacts for dedicated support programs (e.g., smoking cessation) are shared, even if they were already introduced during the assessment phase. | Patient,  Caregivers | - Presentation slides - Recording of the session | Pulmonologist |
| **5- Physical activity and exercise** | The session outlines the health benefits of physical activity, providing guidance on the recommended amount and intensity. It also defines the specific considerations for people with asthma.  Practical advice is given on how to choose appropriate exercises and maintain an active lifestyle. | Patient,  Caregivers | - Presentation slides - Recording of the session | Physiotherapist, |
| **6- Healthy diet** | The session describes the principles of a healthy and balanced diet and how these can be integrated into asthma management.  It promotes behaviors and strategies to support a balanced diet and, where necessary, to assist with BMI reduction. | Patient,  Caregivers | - Presentation slides - Recording of the session | Dietician, |
| **7- Mental and physical well-being** | The session addresses the topic of psychological well-being in its various aspects. It offers practical suggestions for managing and enhancing one’s well-being in daily life and emphasizes the importance of psychological support. Strategies for relaxation are also recommended. | Patient,  Caregivers | - Presentation slides - Recording of the session | Psychologist |
| **8- From now on: Maintaining the results of rehabilitation** | Questions, Feedback, and Looking Ahead  Participants are invited to share any questions, doubts, or concerns regarding the recently completed rehabilitation program, including both the educational and exercise components.  A dedicated section focuses on the continuation and maintenance of the habits acquired during the program, highlighting their importance and introducing monitoring tools to support long-term adherence.  The importance of the follow-up program is also emphasized. | Patient,  Caregivers,  General Practitioner (GP) | - Presentation slides - Recording of the session - Short informational brochure (both printed and available online) | Pulmonologist,  Cardiologist  Physiotherapist, Dietician,  Psychologist |
