# Supplementary material for: Designing an effective pulmonary rehabilitation program for severe asthma
Source: Front Med (Lausanne). 2026 Feb 3;13:1761011. doi: 10.3389/fmed.2026.1761011 (PMC12909588; doi:10.3389/fmed.2026.1761011)
Supplement: Supplementary file 2 [file Table_2.docx]

**Supplemtary table B. Components and description of the exercise program, number of suggested repetitions, and function targeted by training.**

| **Exercise** | **Description** | **N° of repetitions** | **Progression** | **Facilitation** | **Target** |
| --- | --- | --- | --- | --- | --- |
| **Sit to stand** | Starting position: sit on a chair. Stand-up. | 8-12 | Increase number of repetitions, up to 3 series of 8-12.  Use ankle weights or dumbbell.  Use a chair with a lower seat height. | Use upper limbs to stand up or anterior support. | Core, Lower limbs, functional activities. |
| **Step up** | Stand facing the step. Step up with one foot, then bring the other foot up. Step down and repeat. | 8-12 | Increase number of repetitions, up to 3 series of 8-12.  Use ankle weights or dumbbell.  Use a higher step. | Perform the exercise with lateral or anterior support. | Lower extremity ancd core muscle strengthening. |
| **Leg extension** | While seated, extend the lower leg at the knee. | 8-12 | Increase number of repetitions, up to 3 series of 8-12.  Use ankle weight or elastic band. |  | Lower extremity muscle strengthening. |
| **Leg Abduction** | Stand upright, abduct the leg to the side, then return to the starting position. | 8-12 | Increase number of repetitions, up to 3 series of 8-12.  Use ankle weight or elastic band. | Perform the exercise with anterior support. | Lower extremity muscle strengthening,  Balance. |
| **Overhead arm raises** | Perform slow arm raises above shoulder level. | 8-12 | Increase number of repetitions, up to 3 series of 8-12.  Use dumbbell or elastic band. | Perform the exercise in sitting position.  Adapt the exercise according to articular limitations. | Mobility and strengthening of the shoulder girdle and thoracic spine, and thoracic expansion. |
| **Biceps curls** | Biceps curls using dumbbell or elastic band. | 8-12 | Increase number of repetitions, up to 3 series of 8-12.  Increase weight. | Perform the exercise in sitting position.  Use a dumbbell and use one arm at a time. | Mobility and strengthening of the shoulder girdle and upper limbs. |
| **Elastic band lat pull-down** | Hold the resistance band overhead and pull it down towards your chest, squeezing the shoulder blades together. Control the movement back to the starting position. | 8-12 | Increase number of repetitions, up to 3 series of 8-12.  Increase elastic band resistance. | Perform the exercise in sitting position. | Mobility and strengthening of the shoulder girdle, back and upper limbs.  Improve thoracic expansion. |
| **Full-body stretch** | Raise the arms upward in an opening movement, then cross the arms as you lower them toward the feet. | 5 |  | Perform the exercise in sitting position. | Full-body flexibility, relaxation and body awareness. |
